# Editorial: Impacts of drug-induced oxidative stress

**DOI:** 10.3389/fmed.2023.1191864

**Published:** 2023-05-03

**Authors:** Atsushi Umemura

**Affiliations:** Department of Pharmacology, Kyoto Prefectural University of Medicine, Kyoto, Japan

**Keywords:** drug-induced oxidative stress, reactive oxygen species (ROS), NADPH oxidases (NOXs), cancer, antioxidants

## Introduction

Oxidative stress is an important factor in the initiation and development of various human diseases, including cancer. Oxidative stress can be exacerbated by reactive oxygen species (ROS) generated in the mitochondria, peroxisomes, and endoplasmic reticulum. ROS, including superoxide (${\text{O}}_{2}^{-}$), hydrogen peroxide (H_2_O_2_), and the hydroxyl radical (OH), exert pleiotropic effects that influence multiple biological phenomena, including host defense, cellular proliferation, cellular senescence and death, and disease progression.

ROS are generated continuously by enzymatic reactions involving cyclooxygenases, xanthine oxidases, lipoxygenases, NADPH oxidases (NOXs), and the iron-catalyzed Fenton reaction, all of which can be modulated by drugs or other therapies. ROS are also generated by exposure to ultraviolet radiation, heat, and other physical agents. Notably, some cancer therapies, including chemotherapy and radiotherapy, rely on ROS production and oxidative stress to achieve their full potential. On the other hand, drug-induced oxidative stress is also an important cause of adverse events, making ROS generation a double-edged sword.

This Research Topic presents new research exploring different aspects of drug-induced ROS generation, including the identification of new compounds to prevent ROS-mediated damage, the targeted delivery of antioxidants, and the precise detection of intracellular ROS.

## Repurposing established drugs as novel antioxidants

Tenofovir disoproxil fumarate is a widely prescribed antiviral drug, but it is also associated with renal adverse effects, in part due to oxidative stress. Long-term exposure to the drug is associated with both renal failure and hypertension. However, as reported by Nascimento et al. in their paper, “*Treatment with* β*-blocker nebivolol ameliorates oxidative stress and endothelial dysfunction in tenofovir-induced nephrotoxicity in rats*,” co-treatment with nebivolol may alleviate these effects. As a β-adrenergic receptor antagonist, nebivolol is currently used in the treatment of hypertension and cardiovascular disease, but it also acts as an antioxidant, both systemically and in the kidney, and it exhibits remarkable anti-inflammatory and anti-fibrotic properties. The repurposing of this well-established drug has the potential to support the use of tenofovir through its modulation of the NO cascades and its role in maintaining the activity of the renin-angiotensin-aldosterone system.

## Antioxidants to limit side effects

Cisplatin and many other chemotherapeutic agents exert their anti-cancer effects in part by inducing ROS-mediated cell damage in cancer cells. However, the therapeutic use of cisplatin is limited by its irreversible side effects, especially in the kidney.

In their report, “*Isoliquiritin ameliorates cisplatin-induced renal proximal tubular cell injury by antagonizing apoptosis, oxidative stress, and inflammation*,” Pei et al. provide important insights into the protection of kidney tissue from cisplatin-induced oxidative stress. Isoliquiritin, a flavonoid glycoside compound, was found to protect the proximal tubule from cisplatin-induced damage. Although *in vivo* studies are needed, the mechanism of action of the drug involves apoptosis and oxidative stress in addition to inflammation. These findings may ultimately lead to a more effective use of cisplatin in the clinic.

It is clear that drug-induced oxidative stress is a major issue that hinders the optimal use of several important drugs. These reports provide important new tools to protect healthy tissue from the effects of ROS-inducing therapeutic agents.

## Approaches to protecting normal tissue without reducing drug efficacy

The side effects of ROS-inducing drugs such as cisplatin could be mitigated by compounds that reduce ROS levels. Unfortunately, systemic application of ROS scavengers would also lower the therapeutic effect, for example, by decreasing the sensitivity of cancer cells to cisplatin. The development of platforms to target such compounds in the kidney is therefore important, as it would reduce dose-limiting toxicities while maintaining the efficacy of cisplatin for cancer treatment.

## Targeted delivery of these compounds

Importantly, the clinical use of cisplatin may be further enhanced by more precise sub-cellular targeting of protective agents. In particular, several models of cisplatin-induced nephrotoxicity and neurotoxicity have been shown to involve mitochondrial damage, and targeted delivery of antioxidants to mitochondria has been shown to reduce the onset of cisplatin-induced renal cell damage (1). These observations suggest that mitochondrial oxidative damage is a component of the dose-limiting toxicities of cisplatin and that mitochondria are a prime target for ROS scavengers.

Nanoparticle technology may be a useful tool to target relevant drugs to the kidney and mitochondria. Recently, increased attention has been paid to the development of stimulus-responsive nanoparticles, which allow accurate spatiotemporal control of drug release and minimize toxicity by avoiding unwanted effects at non-target sites (2, 3). Thus, nanocarriers activated in the renal environment would help regulate ROS levels to protect the kidney while maintaining cisplatin sensitivity in tumors. In addition, further developments may lead to the use of nanoparticles to regulate ROS levels in intracellular organelles, including mitochondria, and nanoparticles may also be developed that allow selective targeting of other organs or even the selective release of ROS-inducing drugs into cancer cells.

## Precision measurement of intracellular ROS

Because ROS can be both friend and foe in the treatment of cancer and other diseases, accurate measurement of intracellular ROS levels would provide clinicians with an important tool for improving the efficacy and safety of therapeutic agents.

Although the detection of extracellular ROS is reasonably reliable, the detection of intracellular ROS remains challenging due to the presence of other oxidants, such as peroxidases, cytochromes, and other iron- and heme-containing proteins.

Matsumoto et al. have made a major advance in the detection of low levels of ROS by demonstrating the application of peptide-boronic acids to the detection of superoxide. In their article, “*Bortezomib is an effective enhancer for chemical probe-dependent superoxide detection*,” they show that the widely used proteasome inhibitor bortezomib sensitizes the detection of superoxide in both cell-based and cell-free systems (Matsumoto et al.). If the assay system is developed for routine use, bortezomib may be an important tool to improve the detection of ROS.

## Conclusion

In summary, this Research Topic focuses on the complexity of drug-induced oxidative stress. The relationships between ROS and the occurrence and development of various human diseases, including cancer, have been intensively studied, and the importance of ROS for therapeutic drug action has been increasingly appreciated. Although the functions and production of drug-induced ROS continue to be characterized and possible means to ameliorate drug-induced oxidative stress have been proposed, further research is needed.

## Author contributions

AU contributed to conception and wrote the manuscript.
